# Effect of extracellular matrix on testosterone production during *in vitro* culture of bovine testicular cells 

**Published:** 2017-03-15

**Authors:** Vahid Akbarinejad, Parviz Tajik, Mansoureh Movahedin, Reza Youssefi

**Affiliations:** 1 *Young Researchers and Elites Club, Roudehen Branch, Islamic Azad University, Roudehen, Iran; *; 2 *Department of Theriogenology, Faculty of Veterinary Medicine, University of Tehran, Tehran, Iran; *; 3 *Department of Anatomical Sciences, Faculty of Medical Sciences, Tarbiat Modares University, Tehran, Iran.*

**Keywords:** Bovine testicular cells, Extracellular matrix Testosterone

## Abstract

Testosterone is believed to play a significant role in spermatogenesis, but its contribution to the process of spermatogenesis is not completely understood. Given that extracellular matrix (ECM) facilitates differentiation of spermatogonial stem cells (SSCs) during culture, the present study was conducted to elucidate whether testosterone contribute to the permissive effect of ECM on SSCs differentiation. In experiment 1, testosterone production was measured in testicular cells cultured for 12 days on ECM or plastic (control). In experiment 2, testosterone production was assessed in testicular cells cultured on ECM or plastic (control) and exposed to different concentrations of hCG. In experiment 3, the gene expression of factors involved in testosterone production was analyzed. Testosterone concentration was lower in ECM than in the control group in experiment 1 (*p* < 0.05). In experiment 2, testosterone concentration was increased in response to hCG in both groups but cells cultured on ECM were more responsive to hCG than those cultured on plastic (*p* < 0.05). In the experiment 3, qRT-PCR revealed the inhibitory effect of ECM on the gene expression of steroidogenic acute regulatory protein (StAR) (*p *< 0.05). Nevertheless, the expression of LH receptor was greater in ECM-exposed than in unexposed cells (*p* < 0.05). In conclusion, the present study showed that inhibiting the expression of StAR, ECM could lower testosterone production by Leydig cells during *in vitro* culture. In addition, the results indicated that ECM could augment the responsiveness of Leydig cells to hCG through stimulating the expression of LH receptor.

## Introduction

Spermatogenesis is a complex process of cell proliferation and differentiation culminating in production of mature spermatozoa from spermatogonial stem cells (SSCs).^1^^,^^2^ Somatic cells encompassing SSCs, called niche cells, regulate the self-renewal and differentiation of SSCs by production of hormones and growth factors, thereby contributing to the perpetuation of spermatogenesis.^1^

Testosterone, produced by Leydig cells in the testis, is one of the factors that is believed to play an essential role in spermatogenesis^3^^,^^4^ as some men with diminished spermatogenesis experience low levels of circulatory testosterone concentration as well.^5^ Dysfunction of Leydig cells has also been observed to lead to spermatogenic disruption.^6^^,^^7^ In addition, induction of spermatogenesis in mice with gonadotropin deficiency has been reported following treatment with testosterone.^8^ Indeed, three major defects have been determined to ensue testosterone deficiency and/or disruption in testosterone action including disintegration of blood-testis barrier,^9^^-^^12^ blockade in conversion of round to elongated spermatids^13^^-^^15^ and disruption in mature spermatozoa release from Sertoli cells.^13^ Yet the role of testosterone in the process of spermatogenesis is not completely understood and requires to be elucidated.^4^

Testosterone synthesis by Leydig cells is under the influence of luteinizing hormone (LH). This mechanism could also be regulated by the LH analogue, human chorionic gonadotropin (hCG).^16^ Steroidogenic acute regulatory protein (StAR), which is produced in response to LH, plays an essential role in testosterone production through translocating intracellular cholesterol to inner mitochondrial membrane in which the cholesterol side chain would be cleaved leading to production of pregnenolone, the precursor for testosterone.^17^

Extracellular matrix (ECM), composed of macro-molecules with different structural and functional properties, could impact the SSCs function through generation of signals by ECM molecules per se, as well as modulating the other extracellular stimuli, including growth and differentiating factors.^18^^-^^20^ Application of ECM during in vitro culture of cells provides an environment partly resembling the normal tissue^18^^,^^21^^,^^22^ and has been indicated to have permissive effect for differentiation of SSCs.^23^^-^^25^ This phenomenon has been attributed to the impact of ECM on the behavior of niche cells and the interaction between niche and germ cells.^24^ The ECM has been observed to influence the shape and function of Leydig cells.^26^ In addition, ECM has been reported to influence the production of testosterone by Leydig cells.^27^^-^^29^

In the previous studies investigating the effect of ECM on SSCs differentiation, the impact of ECM on the potential contribution of testosterone in terms of SSCs differentiation has not been assessed.^23^^-^^25^ Therefore, the present study was conducted to address whether testosterone contribute to the differentiating effect of ECM on SSCs during in vitro culture.

## Materials and Methods

 Animals and testicular biopsy. Animal Ethics Committee at University of Tehran approved the present study in terms of animal welfare and ethics. Testicular biopsies were obtained from Holstein calves (n = 17; aged 3 to 5 months) as previously described.^23^ In brief, testicular biopsy was performed under sedation with xylazine (0.20 mg kg^-1^; Alfasan, Woerden, Holland) and local anesthesia with lidocaine (Aburaihan Pharmaceutical Co., Tehran, Iran). Following incision, the testicular tissue was obtained and placed into a 15 mL tube containing Dulbecco' minimal essential medium (DMEM; Gibco, Carlsbad, USA) with 10% fetal bovine serum (FBS; Sigma-Aldrich, St. Louis, USA) and antibiotics (100 IU mL^-1^ penicillin and 100 µg mL^-1^ streptomycin; Gibco). Following testicular biopsy, the specimen was transferred on ice to the laboratory within 2 hr.

Experiment 1. Testicular cells (n = 7 cell populations from different calves) were cultured for 12 days and testosterone production was measured on days 6 and 12 of culture.^23^

Experiment 2. Testicular cells (n = 7 cell populations from different calves) were cultured for 7 days. On day 6, hCG (IVF-C^®^; LG Life Sciences, Seoul, South Korea) was added to cultures at concentrations of 0.00, 0.10, 1.00 and 10.00 IU L^-1^ for a one-day period, afterwards testosterone concentration was evaluated.^27^

Experiment 3. Testicular cells (n = 3 cell populations from different calves) were cultured for 12 days and gene expression of luteinizing hormone receptor (LHR), CYP12A1, which codes for a member of the cytochrome P450 superfamily of enzymes contributing to androgen synthesis^30^ and steroidogenic acute regulatory protein (StAR), which is responsible for transferring cholesterol into mitochondria thereby contributing to steroid-genesis,^31^ were evaluated using quantitative real time PCR on days 6 and 12.^23^

Cell isolation. Cell isolation was implemented using a two-step enzymatic isolation procedure.^23^ The testicular tissue was washed three times in DMEM containing antibiotics and was minced into small pieces using a sterile scissor. The minced testicular tissue was incubated in DMEM containing 1 mg mL^-1^ collagenase (Sigma-Aldrich), 1 mg mL^-1^ hyaluronidase (Sigma-Aldrich), 1 mg mL^-1^ trypsin (Sigma-Aldrich) and 5 µg mL^-1^ DNase (Fermentas, Waltham, USA) at 37 ˚C in a shaker incubator with 80 cycles per minute for approximately 60 min. The digested testicular tissue was washed three times with DMEM and the supernatant was disposed after each washing, leading to isolation of seminiferous tubules. During the second step of enzymatic digestion, seminiferous tubules were incubated at 37 ˚C in DMEM containing 1 mg mL^-1^ collagenase, 1 mg mL^-1^ hyaluronidase and 5 µg mL^-1^ DNase until disintegration of the seminiferous tubules and separation of the constituent cells. Individual cells were isolated from the remaining tubule fragments by centrifugation at 30 g for 2 min. Following filtration through 77 and 55 mm nylon filters, the cells were pelleted. The pellet was re-suspended in the DMEM containing antibiotics and 10% knock-out serum replacement (KSR; Gibco).

Cell culture. Wells used for the control group were uncoated. Wells used for the ECM group were coated with ECM gel (Sigma-Aldrich) as the manufacturer indicated. The ECM gel was prepared from Engelbreth-Holm-Swarm (EHS) mouse sarcoma and was composed of laminin as the major component, collagen type IV, heparan sulfate proteoglycan, entactin and other minor components. In the experiments related to assess-ment of gene expression and testosterone concentration, 6 and 24-well plates were used, respectively. In the experiments associated with evaluation of gene expression and testosterone measurement, cells were seeded at concentrations of 1 × 10^6^ and 3 × 10^5^ cells per well containing DMEM with antibiotics and 10% KSR, respectively. The plates were incubated at 37 ˚C in a humidified atmosphere with 5% CO_2_. The medium was replaced with fresh one every three days.^23^

Testosterone concentration analysis. Testosterone concentration was measured by direct, competitive, chemi-luminescence immunoassay kit (DiaSorin, Stillwater, USA).

RNA isolation and quantitative real-time PCR (qRT-PCR). Following trypsinization of the cultured cells, the isolated cells were subjected tototal RNA extraction using Trizol reagent (Fermentas). The extracted RNA was treated with DNase (Fermentas) to eliminate DNA contamination. The concentration of RNA was determined using UV spectrophotometry (Eppendorff, Hamburg, Germany). The cDNAs were synthesized from 500 ng of RNA by oligo (dT) primers using RevertAid™ first strand cDNA synthesis kit (Fermentas). Primers for genes of interest are shown in Table 1. The PCRs were performed using master mix and SYBR Green I (Fermentas) in a thermal cycler (Applied Biosystems, California, USA). The PCR program started with an initial melting cycle for 5 min at 95 ˚C to activate the polymerase, followed by 40 cycles of melting (30 sec at 95 ˚C), annealing (30 sec at 58 ˚C) and extension (30 sec at 72 ˚C). The quality of the PCR reactions was confirmed by melting curve analyses. For each sample, the reference gene (β-actin) and target gene were amplified in the same run. The target genes were normalized to the reference gene. The mean target gene threshold cycle (Ct) and mean exogenous control (β-actin) Ct for each sample were calculated from duplicate wells. The target gene Ct of the control was subtracted from the Ct of target gene, resulting in ∆Ct. In each experiment, the Ct of time-point 0 sample was considered as calibrator. Subsequently, the ∆Ct of sample was then subtracted from the ∆Ct of calibrator, resulting in the ∆∆Ct, which was used for calculation of the relative amounts of target gene expression for each sample.^32^

**Table 1:** Primer sequences used for qRT-PCR

**Gene**	**Forward primer (5´-3´)**	**Reverse primer (5´-3´)**
***β-actin***	TCG CCC GAG TCC ACA CAG	ACC TCA ACC CGC TCC CAA G
***Lhr *** a	TGACCATGGCCCGTCTAAAA	TACTACCCAAAGCAATTTATAGATTCAATG
***Cyp17a1***	CACCGATATTATCAGAAACCC	ATTGGTGATGGACTCAAAGG
***Star *** b	GTGGAATCCCAATGTCAAGG	TGATGACCGTGTCTTTTCCA

a LH receptor;

b Steroidogenic acute regulatory protein.

Statistical analysis. Initially, datasets were tested for normal distribution using Kolmogorov-Smirnov test (univariate procedure). Data associated with testosterone concentration in experiment 1 were log (base 10) transformed prior to analysis because of the lack of normal distribution. Data were analyzed using mixed procedure including random and repeated statements in the model to specify co-variation between and within calves respectively.^33^ In addition, LSMEANS statement was used to perform multiple comparisons. All analyses were conducted in SAS (version 9.2; SAS Institute, Cary, USA).Data are presented as mean ± SD. Differences with p < 0.05 were considered significant.

## Results

Experiment 1. Testosterone concentration was higher in the control than ECM group on days 6 and 12 of culture (p < 0.05). However, the production of testosterone was not different between days 6 and 12 in the control and ECM groups (p > 0.05; Table 2).

**Table 2:** Testosterone concentration (ng mL^-1^) in control and extracellular matrix (ECM) groups on Days 6 and 12 of culture. Data are presented as mean ± SD

**Gene**	**Day 6**	**Day 12**
**Control**	3.50 ± 1.18^a^	3.72 ± 1.70^a^
**ECM**	1.64 ± 0.48^b^	1.94 ± 0.77^b^

ab Different superscripts indicate significant differences within each column (*p *< 0.05).

Experiment 2. In the control group, testosterone concentration in cells cultured with 10.00 ng mL^-1^ hCG was greater than that in cells cultured with 0.00 and 0.10 IU mL^-1 ^of hCG (p < 0.001). However, the concentration of testosterone did not differ among 0.00, 0.10 and 1.00 IU mL^-1 ^concentrations of hCG and between 1.00 and 10.00 IU mL^-1 ^concentrations of hCG (p > 0.05; Table 3). In ECM group, testosterone concentration in 10.00 IU mL^-1 ^hCG group was greater than that in 0.00, 0.10 and 1.00 IU mL^-1 ^hCG groups (p < 0.05). In addition, testosterone concentration was higher in 1.00 IU mL^-1 ^hCG group than 0.00 and 0.10 IU mL^-1^ hCG groups (p < 0.05). Testosterone concentration was not different between 0.00 and 0.10 IU mL^-1 ^hCG groups (p > 0.05; Table 3).

**Table 3:** Testosterone concentration (ng mL^-1^) in control and extracellular matrix (ECM) groups following exposure by different levels of hCG. Data are presented as mean ± SD

**Groups**	**hCG concentration (IU L** ^-1^ **)**			
	**0.00**	**0.10**	**1.00**	**10.00**
**Control**	3.64 ± 0.65^a^^A^	3.73 ± 0.89^a^^A^	4.10 ± 0.74^ab^^A^	4.49 ± 0.70^b^^A^
**ECM**	2.43 ± 0.44^a^^B^	2.62 ± 0.43^a^^B^	3.05 ± 0.39^b^^B^	3.47 ± 0.38^c^^B^

abc Different lowercase superscripts indicate significant differences within each row (*p *< 0.05).

AB Different uppercase superscripts indicate significant differences within each column (*p *< 0.05).

**Fig. 1:**
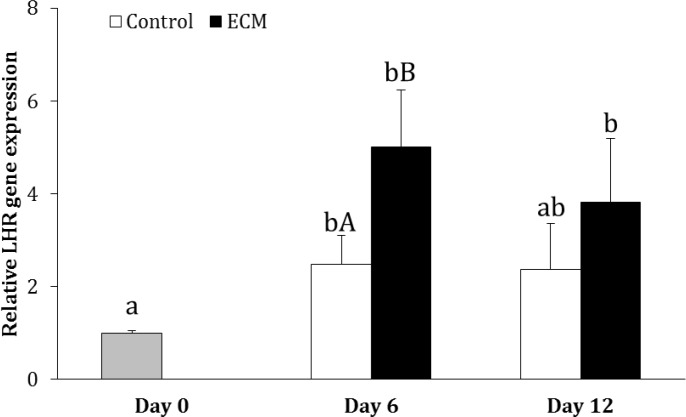
Relative gene expression of LHR in the control and ECM groups (n = 3) on days 6 and 12. Lowercase letters (a and b) indicate significant difference within groups between different timepoints (*p *< 0.05). Uppercase letters (A and B) indicate significant difference between two experimental groups at the specified timepoint (*p* < 0.05

Experiment 3. In the control group, the gene expression of LHR on day 6 (2.47 ± 0.62 fold) was higher than that on day 0 (p < 0.05); however, LHR expression on day 12 was not different from days 0 and 6 (p > 0.05). In ECM group, the expression of LHR on days 6 (5.00 ± 1.23 fold) and 12 (3.81 ± 1.38 fold) was greater than that on day 0 (p < 0.01), but it did not differ between days 6 and 12 (p > 0.05). On day 6, the gene expression of LHR was higher in ECM than the control group (p < 0.05), but it was not different between two groups on day 12 of culture (p > 0.05; Fig. 1).

**Fig. 2:**
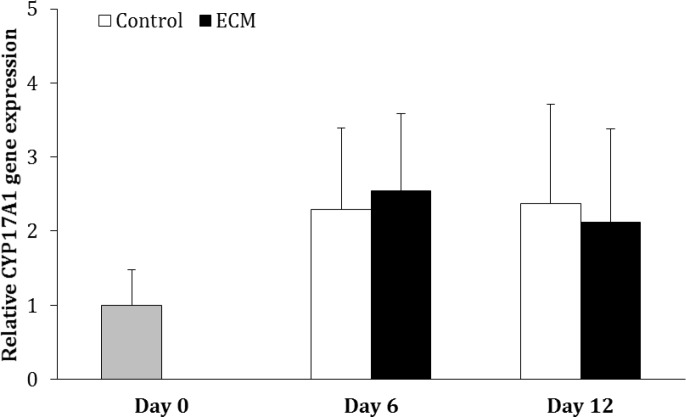
Relative gene expression of CYP17A1 in the control and ECM groups (n = 3) on days 6 and 12. No significant differences were noted among the groups

The gene expression of CYP12A1 did not change over the course of culture in the control and ECM groups (p > 0.05) and it was not different between two groups on days 6 and 12 (p > 0.05; Fig. 2).

**Fig. 3:**
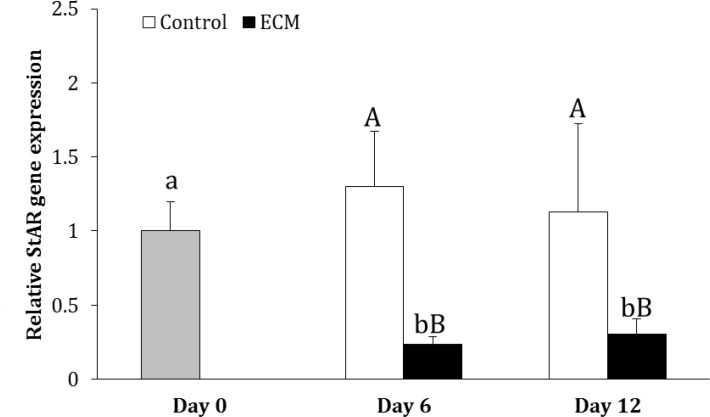
Relative gene expression of StAR in the control and ECM groups (n = 3) on days 6 and 12. Lowercase letters (a and b) indicate significant difference within groups between different time points (*p* < 0.05). Uppercase letters (A and B) indicate significant difference between two experimental groups at the specified time point (*p* < 0.05

In the control group, the expression of StAR did not change during culture (p > 0.05). In ECM group, however, the expression of StAR was 77.00% and 70.00% lower on days 6 and 12, respectively, as compared to day 0 (p < 0.05). The expression of StAR in ECM group was lower than that in the control group on days 6 and 12 (p < 0.05; Fig. 3).

## Discussion

The present study revealed that ECM decreased the production of testosterone by Leydig cells during in vitro culture. Culturing testicular germ cells of rat on ECM for 22 days, Lee et al. reported differentiation of germ cells up to haploid cells.^24^ The same observation was indicated when ECM was applied for culture of human testicular germ cells.^25^ More recently, culturing bovine SSCs on ECM for a 12-day period, Akbarinejad et al. found evidence for the initiation of SSCs differentiation. This phenomenon was not observed following culture of SSCs on plastic.^23^ On the other hand, giving mice flutamide, an androgen receptor antagonist, Shetty et al. observed stimulation of differentiation in spermatogonia in mice.^34^ The same phenomenon was detected when GnRH antagonist, decreasing intratesticular testosterone concentration, was used.^34^ Further, it was revealed that combination of flutamide and GnRH antagonist intensified spermatogonial differentiation, whereas combination of testosterone with GnRH antagonist inhibited spermatogonial differentiation.^34^ Taken together, it was concluded that testosterone played an inhibitory role in differentiation of spermatogonia through binding to androgen receptor,^34^ which was substantiated by a further study.^35^ Moreover, using a Sertoli-cell androgen receptor knockout model, Zhou et al. demonstrated the inhibitory effect of androgen receptor of Sertoli cells in spermatogonial differentiation.^36^ Therefore, inhibition of testosterone synthesis by Leydig cells might be one of the mechanisms whereby ECM molecules facilitate SSCs differentiation.^23^^-^^25^ In our previous study, we indicated that ECM molecules mediated their differentiative effect on SSCs through upregulation of KIT ligand.^23^ Further studies are warranted to elucidate the other potential mechanisms contributing to permissive effect of ECM on SSCs differentiation.

Evaluating the effect of different types of proteins composing ECM, Diaz et al. indicated that laminin did not influence testosterone production, however, collagen type IV and fibronectin decreased the production of testosterone by Leydig cells.^27^ Further, investigating the downstream mechanisms whereby collagen type IV reduces testosterone production, Diaz et al. found that the inhibitory effect of collagen type IV was mediated through activating extracellular signal-regulated kinase and decreasing cAMP production and StAR expression.^37^ The gene expression of StAR has also been revealed to be lower in the ECM-exposed cells compared to unexposed ones in the present study. Collagen type IV was a part of the ECM used in the present study. Therefore, the lower concentration of testosterone in ECM-coated plates could have been originated from inhibition of StAR expression by collagen type IV.

Although testosterone production was increased in response to addition of hCG in ECM-exposed and unexposed cells and it was higher at all concentrations of hCG in the control than ECM group, it was shown that cells cultured on ECM-coated plates were more responsive to hCG compared to those cultured on uncoated plates. Gene expression analysis in the present study showed that ECM increased LH receptor expression, which probably contributed to greater responsiveness of Leydig cells cultured on ECM to hCG. Proteoglycans have been shown to modulate testosterone production by Leydig cells in rat.^28^^,^^29^ Factors neutralizing heparan sulfate proteoglycans have been observed to decrease testosterone production in LH-stimulated Leydig cells, suggesting that heparan sulfate proteoglycans have positive impact on testosterone production through modulating LH receptor.^29^ On the other hand, no significant effect of collagen type IV on the specific binding of hCG to LH receptor was observed in rat.^37^ Accordingly, the greater responsiveness of Leydig cells to hCG in ECM than control group could have resulted from the modulating effect of heparan sulfate proteoglycan on LH receptor.

Indeed, different types of ECM are composed of a great variety of molecules with various functional and structural properties, which give each type of ECM specific capabilities for regulating the function and development of cells.^18^ In the present study, it was revealed that a combination of laminin, collagen type IV, heparan sulfate proteoglycan and entactin, which were derived from Engelbreth-Holm-Swarm mouse sarcoma, modulated factors contributing to testosterone synthesis and thus impacted testosterone production. Further studies are required to investigate the specific role of each ECM molecule as well as their interactive effect during the process of testosterone synthesis. Furthermore, the downstream mechanisms whereby ECM molecules influence testosterone production need to be elucidated.

In conclusion, the present study revealed that ECM decreased testosterone production by Leydig cells, through inhibiting StAR expression. Considering the permissive effect of ECM on SSCs differentiation, inhibition of testosterone production was probably one of the mechanisms whereby ECM facilitated SSCs differentiation. Additionally, it was shown that Leydig cells cultured on ECM were more responsive to hCG, which could have stemmed from the stimulatory effect of ECM molecules on LH receptor expression.
